# Integrating Rapid Diabetes Screening Into a Latinx Focused Community-Based Low-Barrier COVID-19 Testing Program

**DOI:** 10.1001/jamanetworkopen.2022.14163

**Published:** 2022-05-26

**Authors:** Andrew D. Kerkhoff, Susana Rojas, Douglas Black, Salustiano Ribeiro, Susy Rojas, Rebecca Valencia, Jonathan Lemus, Joselin Payan, John Schrom, Diane Jones, Simone Manganelli, Shalom Bandi, Gabriel Chamie, Valerie Tulier-Laiwa, Maya Petersen, Diane Havlir, Carina Marquez

**Affiliations:** 1 Division of HIV, Infectious Diseases and Global Medicine, Zuckerberg San Francisco General Hospital and Trauma Center, University of California, San Francisco; 2 San Francisco Latino Task Force–Response to COVID-19, San Francisco, California; 3 Bay Area Phlebotomy and Laboratory Services, San Francisco, California; 4 Unidos en Salud, San Francisco, California; 5 Division of Epidemiology and Biostatistics, School of Public Health, University of California, Berkeley

## Abstract

**Importance:**

Community-based COVID-19 testing and vaccination programs play a crucial role in mitigating racial and ethnic disparities in COVID-19 service delivery. They also represent a platform that can be leveraged to expand access to testing for chronic diseases, including diabetes, that disproportionately affect the Latinx community and other marginalized communities.

**Objective:**

To evaluate outcomes associated with a diabetes testing strategy designed to reach low-income Latinx persons by leveraging COVID-19 testing infrastructure and community trust developed during the COVID-19 pandemic.

**Design, Setting, and Participants:**

This health care improvement study was conducted from August 1 to October 5, 2021, at an outdoor, community-based COVID-19 testing site at a transport hub in the Mission Neighborhood in San Francisco, California. Because the program was designed to expand access to diabetes screening to the local community, all individuals presenting for on-site testing were eligible. Data were analyzed in November 2021.

**Interventions:**

Integration of rapid, point-of-care hemoglobin A_1c_ screening as a testing option in an existing low-barrier COVID-19 testing program.

**Main Outcomes and Measures:**

Evaluation was guided by the Reach, Effectiveness, Adoption, Implementation and Maintenance (RE-AIM) framework and utilized programmatic data and structured surveys among clients and staff.

**Results:**

Of 6631 individuals tested (median [IQR] age 39.3 [29.7-51.3] years; 3417 [52.3%] female, 4348 [65.6%] Latinx), 923 (13.9%) underwent hemoglobin A_1c_ testing with or without COVID-19 testing and 5708 (86.1%) underwent COVID-19 testing only. Individuals tested for diabetes were more likely to be Latinx (763 of 923 individuals [82.7%] who underwent testing were Latinx vs 3585 of 5708 [62.8%] not undergoing testing), have an annual household income of less than $50 000 (450 individuals [81.2%] vs 2409 individuals [66.0%]), and not have health insurance (381 individuals [47.2%] vs 1858 individuals [39.9%]), and 206 (48.0%) had never tested for diabetes before. Overall, 313 (33.9%) and 113 (12.2%) individuals had prediabetes and diabetes, respectively; only 141 of 354 of these individuals (39.8%) had a primary care clinician whom they had seen in the prior 12 months, which was lower among Latinx individuals (113 of 307 individuals [36.8%] vs 28 of 47 [59.6%]). Acceptability of the rapid testing program was high—98% were satisfied with their visit and 96% said they would return for future services; key factors underpinning acceptability included friendly staff, efficiency, and a convenient location.

**Conclusions and Relevance:**

In this health care improvement study conducted within an existing community-based COVID-19 testing program, integrating rapid testing for diabetes was feasible, reached low-income Latinx individuals, and identified many persons with prediabetes and diabetes, most of whom lacked access to services in formal health care settings. Leveraging pandemic-related public health responses represents an important opportunity for engaging socioeconomically disadvantaged populations into care for diabetes.

## Key Points

**Question:**

Is integrating rapid diabetes screening into a community-based COVID-19 testing site feasible and effective in reaching socioeconomically disadvantaged Latinx persons without health care?

**Findings:**

In this health care improvement study of 6631 participants presenting for COVID-19 testing, 923 (13.9%) underwent hemoglobin A_1c_ testing, of which 313 (34%) and 113 (12%) had prediabetes and diabetes, respectively. Persons reached were mostly Latinx (83%), low-income, had not previously been tested for diabetes, and were not engaged in formal health care services.

**Meaning:**

These results suggest that leveraging community COVID-19 infrastructure and partnerships has the potential to address disparities in diabetes.

## Teaser

This health care improvement study examines the effectiveness of integrating hemoglobin A_1c_ testing for diabetes and prediabetes into a low-barrier COVID-19 testing site in a Latinx community in a large US city.

## Introduction

COVID-19 has laid bare and amplified systemic inequities in health outcomes endured by Latinx and other historically marginalized communities in the US.^1,2^ It has also highlighted the importance of community partnerships and delivery of services outside of traditional brick-and-mortar health care facilities to mitigate these inequities, especially for people who face access-related barriers to health care.^3,4,5,6,7^ Community-based COVID-19 testing infrastructure and community partnerships can be leveraged to address additional health inequities, including chronic diseases such as diabetes, but to date such a strategy has received little attention.

As with COVID-19, Latinx persons in the US are disproportionately affected by diabetes. Latinx persons have a 65% greater risk of being diagnosed with type 2 diabetes than non-Latinx White individuals, and once diagnosed they are more likely to experience diabetes-related complications including vision loss, kidney failure, and death.^8,9,10,11^ Barriers to diabetes and COVID-19 care and engagement among socioeconomically disadvantaged Latinx persons are similar and include a lack of health insurance or primary care clinician, unavailability of culturally and linguistically concordant services, immigration concerns and fear of deportation, and the direct and indirect costs associated with attending appointments.^12,13,14^ Diabetes education and testing programs at convenient community sites such as churches or flea markets can overcome key trust and access-related barriers among low-income and immigrant Latinx communities, but these programs are typically event-based, and standing community-based multidisease testing programs remain largely unexplored.^15,16,17^ Integrating rapid diabetes screening into community-based COVID-19 testing sites has the potential to improve the prevention, diagnosis, and entry into care for diabetes.

Since April 2020, the Unidos en Salud community-academic–public health partnership between the San Francisco Latino Task Force–Response to COVID-19 (LTF) and University of California, San Francisco has been providing low-barrier COVID-19 testing, vaccination, and social support services tailored to the needs of the Latinx community in San Francisco.^18^ During this period, Unidos en Salud has established high levels of trust among a socioeconomically disadvantaged community that has many unmet needs. We hypothesized that a theory-informed, multidisease rapid testing strategy could leverage this trust and our existing COVID-19 testing infrastructure situated outside of the formal health care system to identify a large number of Latinx persons with prediabetes and diabetes who may face substantial barriers to care engagement. This study evaluated the reach, acceptability, feasibility, and effectiveness of this novel strategy to integrate rapid hemoglobin A_1c_ testing into an established community-based COVID-19 testing program.

## Methods

### Study Setting and COVID-19 Testing and Vaccination Program

The outdoor, neighborhood-based Unidos en Salud COVID-19 testing and vaccination program is located in the Mission District, San Francisco, California. The Mission District is an important cultural and commercial hub for Latinx people in the San Francisco Bay Area and is highly racially, ethnically, and economically diverse.^19^ The Unidos en Salud neighborhood COVID-19 testing and vaccination program has previously been described in detail.^20,21,22^ In brief, the design of the low-barrier site was theory-informed in order to overcome barriers to health care engagement commonly faced by socioeconomically disadvantaged, Latinx persons to optimize access and uptake of COVID-19 services. Vaccination and testing services were colocated adjacent to a busy transportation hub and open 4 days a week, including weekend days. Individuals could either show up for testing without prior notice (referred to as *drop-in appointments*) or register online, and there was no requirement to show identification or to prove residency or health insurance status. The site was culturally tailored and site staff were predominantly monolingual or bilingual Spanish-speaking; many were local community members.

### Ethics Statement

The University of California, San Francisco Committee on Human Research determined that the study met criteria for public health surveillance and was exempt from institutional review board oversight. All survey respondents provided written informed consent in their preferred language. This study is reported in accordance with the Standards for Quality Improvement Reporting Excellence (SQUIRE) reporting guideline.

### Multidisease Rapid Testing Program for Chronic Diseases

Beginning August 1, 2021, we began offering rapid hemoglobin A_1c_ and rapid HIV antibody testing at the Unidos en Salud COVID-19 testing program as part of a health care improvement study; this directly coincided with the arrival of the COVID-19 Delta variant in San Francisco. At the time of registration, individuals were asked whether they wanted to undertake COVID-19 testing, HIV testing, and/or hemoglobin A_1c_ testing. For those wanting COVID-19 testing, laboratory assistants performed a bilateral anterior nasal swab, and their status was determined using a rapid COVID-19 antigen test (Abbott Diagnostics). Fingerstick whole blood was collected by laboratory assistants for individuals who agreed to hemoglobin A_1c_ or HIV antibody testing. Hemoglobin A_1c_ levels were determined using a A_1C_ whole blood test (PTS Diagnostics). All diagnostic tests were performed at the point-of-care according to manufacturer instructions. Clients were provided results disclosure for all tests within 1 hour of testing via secure messaging; for those found to have COVID-19, prediabetes or diabetes, or HIV, a Spanish-speaking bilingual community health worker (CHW) disclosed the results of the test, provided brief health education, and facilitated linkage to primary care. Health education included information about prediabetes or diabetes pathophysiology, brief counseling on lifestyle modifications, and counseling for all patients on the need for confirmatory testing in a primary care clinic. Clients with diabetes who were uninsured were directly referred to a community health clinic for a new patient appointment to establish primary care; clients with diabetes who were insured and had an established primary care clinic but were out of care were provided coaching from a CHW to reengage in care. Uninsured clients with prediabetes were provided coaching on how to call and request primary care through the San Francisco Health Network. All clients with prediabetes and diabetes who had an established primary care clinician and were engaged in care were encouraged to schedule a follow-up visit to discuss their results.

### Evaluation of the Unidos en Salud Multidisease Testing Project

We utilized the Reach, Effectiveness, Adoption, Implementation and Maintenance (RE-AIM) framework to evaluate our strategy of integrating rapid hemoglobin A_1c_ testing into existing community COVID-19 testing infrastructure.^23^ Reach was defined as the number and characteristics of persons who underwent rapid hemoglobin A_1c_ testing. To understand representativeness of those reached by the multitest rapid testing strategy, baseline characteristics were compared according to hemoglobin A_1c_ testing status. Preliminary effectiveness was defined by the number and proportion of persons with diabetes either newly diagnosed and/or not engaged in care. We also evaluated the proportion of clients who received diabetes related counselling, and in a random sample of clients with an A_1C_ level of 6.5% or higher, we evaluated the proportion of clients with diabetes linked to primary care. Implementation outcomes assessed included acceptability of multidisease testing strategy among clients and perceived feasibility of the strategy among Unidos en Salud staff. Because this was a single-site study, adoption and maintenance were not evaluated.

### Statistical Analysis

The present analysis was restricted to adults aged 18 years or older and focused on integration of rapid hemoglobin A_1c_ testing; therefore, it excluded those who only underwent HIV testing. Data were administratively censored on October 5, 2021, corresponding to a 2-month evaluation period. Hemoglobin A_1c_ results were transformed from a continuous variable into an ordinal variable classified as normal (below 5.7%), prediabetes (5.7%-6.49%), and diabetes (6.5% or higher).^24^ Clients undertook surveys at 2 time points. Participants selected their race and ethnicity while completing the electronic testing registration form, and prior to testing they also completed a structured survey that captured additional demographics (including self-identifying for gender), socioeconomic characteristics, medical history, health knowledge, and health care–seeking behaviors. Following completion of testing, clients were also asked to complete an additional structured survey, which evaluated their experience at the site and included measures of acceptability. On October 11 and 12, 2021, Unidos en Salud staff members were asked to complete a brief structured survey, which included a validated tool^25^ to assess perceived feasibility of the multidisease testing strategy. To assess linkage to primary care, a CHW systematically contacted a random sample of clients with diabetes (approximately 20%), testing between August 1 and October 5, 2021, to determine whether they had been linked to primary care services.

We used descriptive statistics to characterize participants. As our diabetes screening strategy and the broader community-based testing program was designed and tailored for Latinx community members, all outcomes, aside from representativeness of clients reached with hemoglobin A_1c_ testing, were undertaken overall and according to clients’ ethnicity (Latinx [self-identifying Latinx/Hispanic and/or American Indian from Central or South America] vs non-Latinx) to evaluate whether the strategy reached Latinx individuals. Wilcoxon rank-sum, Fisher exact, and χ^2^ tests were applied as appropriate to compare characteristics. A cascade-of-care analysis was undertaken to visualize care engagement status among those with prediabetes and diabetes, respectively. All analyses were undertaken using Stata version 17.0 (Stata Corp). All statistical tests were 2-sided at α = .05.

## Results

### Reach

**Figure 1.**
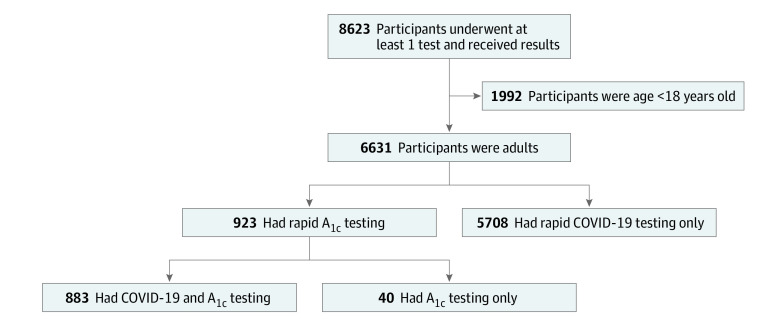
Study Flow Diagram

Overall, there were 6631 unique adults tested for COVID-19 and/or diabetes between August 1 and October 5, 2021 (Figure 1). Clients had a median (IQR) age of 39.3 (29.7-51.3) years; 3417 participants (52.3%) identified as female, 4348 (65.6%) self-identified as Latinx/Hispanic or American Indian from Central of South America, and 2859 (68.0%) had an annual household income less than $50 000 per year (Table 1). For health care characteristics, 2239 participants (41.0%) did not have health insurance, and 2794 (51.1%) did not have a primary care clinician; 5331 (88.0%) had received at least 1 COVID-19 vaccination.

**Table 1.** Baseline Characteristics of Clients According to Rapid Hemoglobin A_1c_ Testing Status

Characteristic	Overall (N = 6631)	Underwent hemoglobin A_1c_ testing (n = 923)	Did not undergo hemoglobin A_1c_ testing (n = 5708)	*P* value^a^
Age, y				
Median (IQR)	39.3 (29.7-51.3)	44.8 (35.7-55.6)	38.5 (29.0-50.4)	<.001
18-30	1893 (28.6)	142 (15.4)	1751 (30.7)	<.001
31-50	3054 (46.1)	468 (50.7)	2586 (45.3)	
51-64	1187 (17.9)	218 (23.6)	969 (17.0)	
≥65	497 (7.5)	95 (10.3)	402 (7.0)	
Gender				
Male	3060 (46.8)	407 (44.3)	2653 (47.2)	.14
Female	3417 (52.3)	505 (55.0)	2912 (51.8)	
Nonbinary	62 (1.0)	6 (0.7)	56 (1.0)	
Ethnicity or race^b^				
African American/Black	222 (3.4)	14 (1.5)	208 (3.6)	<.001
American Indian or Alaska Native	36 (0.5)	2 (0.2)	34 (0.6)	
Asian	537 (8.1)	28 (3.0)	509 (8.9)	
Latinx	4348 (65.6)	763 (82.7)	3585 (62.8)	
American Indian from Central of South America	424 (6.4)	66 (7.2)	358 (84.4)	
Latinx/Hispanic	3924 (59.2)	697 (75.5)	3227 (56.5)	
Pacific Islander or Native Hawaiian	26 (0.4)	4 (0.4)	22 (0.4)	
White	997 (15.0)	49 (5.3)	948 (16.6)	
Other^c^	465 (7.0)	63 (6.8)	402 (7.0)	
Occupation				
Food/beverage, health care	1227 (22.3)	197 (25.4)	1030 (21.7)	<.001
Tradesperson, cleaning, personal services	650 (11.8)	145 (18.7)	505 (10.7)	
Education	423 (7.7)	54 (7.0)	369 (7.8)	
Finance, sales, and technology	491 (8.9)	21 (2.7)	470 (9.9)	
Student	240 (4.4)	19 (2.5)	221 (4.7)	
Retired/homemaker	281 (5.1)	41 (5.3)	240 (5.1)	
Unemployed	666 (12.1)	115 (14.8)	551 (11.6)	
Other^c^	1182 (21.4)	138 (17.8)	1044 (22.0)	
Annual household income, $				
<50 000	2859 (68.0)	450 (81.2)	2409 (66.0)	<.001
50 000-100 000	802 (19.1)	79 (14.3)	723 (19.8)	
>100 000	541 (12.9)	25 (4.5)	516 (14.1)	
Health insurance				
Yes	3221 (59.0)	426 (52.8)	2795 (60.1)	<.001
No	2239 (41.0)	381 (47.2)	1858 (39.9)	
Primary care clinician				
Yes	2676 (48.9)	372 (46.2)	2304 (49.3)	.10
No	2794 (51.1)	433 (53.8)	2361 (50.6)	
Vaccinated against COVID-19^d^				
Yes	5331 (88.0)	735 (90.5)	4596 (87.6)	.02
No	728 (12.0)	77 (9.5)	651 (12.4)	

^a^ *P* value represents Wilcoxon rank-sum test for comparison of medians or χ^2^ or Fisher exact test for comparison of proportions.

^b^ Ethnicity was self-defined by participants.

^c^ Other included participants that did not self-identify with one of the above categories.

^d^ Received at least 1 shot.

Of all clients tested, 923 (13.9%) underwent rapid hemoglobin A_1c_ testing, the large majority (95.7%) of whom were tested for both COVID-19 and hemoglobin A_1c_ level, while 5708 (86.1%) declined hemoglobin A_1c_ testing and were tested for COVID-19 only (Figure 1). Compared with clients who declined diabetes testing, persons who underwent diabetes testing tended to be older (median [IQR] age, 44.8 [35.7-55.6] vs 38.5 [29.0-50.4] years), were more likely be Latinx (763 of 923 individuals [82.7%] who underwent testing were Latinx vs 3585 of 5708 [62.8%] not undergoing testing), have a household income less than $50 000 per year (450 individuals [81.2%] vs 2409 individuals [66.0%]), and not have health insurance (381 individuals [47.2%] vs 1858 individuals [39.9%]) (Table 1). Notably, among clients tested for diabetes, 176 (47.1%) had little-to-no preexisting diabetes-specific knowledge, only 129 (30.1%) had been previously tested for diabetes, and 27 (6.4%) had previously been diagnosed with diabetes; Latinx clients were less likely to report prior diabetes testing (eTable 1 in the Supplement).

The large majority of clients learned about the Unidos en Salud testing site by word of mouth from colleagues, friends, or family members (2780 [49.3%]), or from passing by the site (1508 [26.8%])—small differences were observed by ethnicity (Table 2). Clients reported several factors affecting their decision to get tested at the Unidos en Salud site, including its convenient location (2052 [38.5%]), free services (873 [16.4%]), and a recommendation from a trusted friend, colleague, or family member (641 [12.0%]) (Table 2); convenience was the most important factor regardless of ethnicity, but was more important to non-Latinx clients (940 of 1939 [50.1%] vs 1112 of 3697 [32.3%]), while multidisease testing was more important to Latinx clients (350 of 3697 [10.2%] vs 78 of 1939 [4.2%]) (Table 2). Among clients receiving hemoglobin A_1c_ testing, the most common reasons for getting tested for diabetes were that clients “might as well since they were already getting tested COVID-19,” which was slightly more common among Latinx clients (305 of 3697 [54.0%] vs 52 of 1939 [50.5%]), and because they had either not been tested recently or had never been tested for diabetes, which was slightly more common among Latinx clients (102 [18.1%] vs 16 [15.5%]) (Table 2).

**Table 2.** Factors Affecting Multidisease Testing Uptake at the Unidos en Salud Neighborhood Site According to Latinx Ethnicity

Factor	Overall (n = 5636)	Latinx^a^ (n = 3697)	Non-Latinx (n = 1939)	*P* value^b^
Ways clients learned about site				
Heard about it from a friend, family member, or coworker	2780 (49.3)	1818 (49.2)	962 (49.6)	<.001
Passed by the site	1508 (26.8)	1048 (28.4)	460 (23.7)	
Received a text notifying them about testing at this site	71 (1.3)	53 (1.4)	18 (0.9)	
Found out about it when they came to get a COVID-19 vaccine	193 (3.4)	133 (3.6)	60 (3.1)	
Saw a flyer/billboard for the site	222 (3.9)	170 (4.6)	52 (2.7)	
Saw it on social media	168 (3.0)	103 (2.8)	65 (3.4)	
Saw it in the news (newspaper, TV, radio)	78 (1.4)	41 (1.1)	37 (1.9)	
Other	616 (10.9)	331 (9.0)	285 (14.7)	
Primary reason for choosing to get tested at site				
Convenient—close to home or work	2052 (38.6)	1112 (32.3)	940 (50.1)	<.001
Free services	873 (16.4)	606 (17.6)	267 (14.2)	
Invited by someone they trusted	641 (12.0)	416 (12.1)	225 (12.0)	
Multidisease testing available	428 (8.0)	350 (10.2)	78 (4.2)	
Fast and easy registration	354 (6.7)	233 (6.8)	121 (6.5)	
Bilingual staff	148 (2.8)	131 (3.8)	17 (0.9)	
Prior good experience at site	93 (1.8)	65 (1.9)	28 (1.5)	
No ID or documentation requirements	69 (1.3)	34 (1.0)	35 (1.9)	
Discretion/privacy	60 (1.1)	35 (1.3)	14 (0.8)	
Unable to get tested elsewhere—no insurance and/or a primary care clinician	65 (1.2)	46 (1.3)	19 (1.0)	
Other	542 (10.2)	409 (11.9)	133 (7.1)	
Primary reason for getting tested for diabetes^c^				
Already getting tested for COVID-19, might as well	357 (53.4)	305 (54.0)	52 (50.5)	.02
Never/not recently tested	118 (17.8)	102 (18.1)	16 (15.5)	
Concerned about symptoms that may be diabetes-related	57 (8.5)	49 (8.7)	8 (7.8)	
Encouraged by friend/loved one	59 (8.8)	44 (7.8)	15 (14.6)	
Someone they know has diabetes	46 (6.9)	43 (7.6)	3 (2.9)	
Known diabetes, want to check hemoglobin A_1c_ level	31 (4.6)	22 (3.9)	9 (8.7)	

Abbreviation: ID, identification.

^a^ Latinx includes all persons who self-defined as either Latinx/Hispanic or American Indian from Central or South America.

^b^ *P* value represents χ^2^ or Fisher exact test for comparison of proportions.

^c^ Responses limited to those who underwent rapid hemoglobin A_1c_ testing (668 participants).

### Testing Outcomes

Among all persons who underwent rapid hemoglobin A_1c_ testing, 497 (53.9%) had normal hemoglobin A_1c_ levels, while 313 (33.9%) and 113 (12.2%) individuals met criteria for prediabetes and diabetes, respectively. There was a higher prevalence of prediabetes (34.6% vs 30.6%) and diabetes (12.7% vs 10.0%) among Latinx clients compared with non-Latinx clients; Latinx clients accounted for 84.4% of prediabetes and 85.8% of diabetes diagnoses, respectively. Of clients completing the survey, 41 of 149 (27.5%) and 22 of 55 (40.0%) of those with prediabetes and diabetes, respectively, stated that they had been previously tested for diabetes, while only 15 of 54 (27.9%) of those with diabetes had a prior diabetes diagnosis.

**Figure 2.**
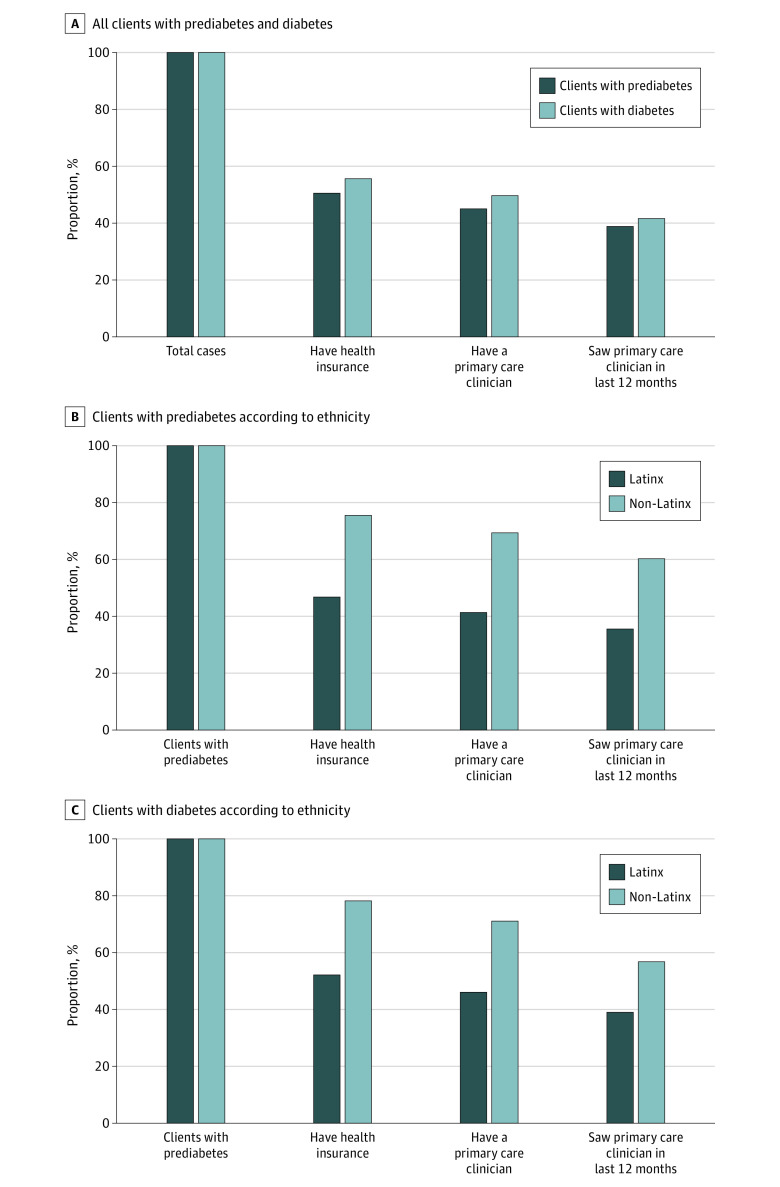
Cascade-of-Care Analysis Among Clients Attending the Unidos en Salud Community-Based Multidisease Testing Site Identified as Having Prediabetes or Diabetes

The cascade-of-care engagement for clients with prediabetes and diabetes is shown in Figure 2. Overall, only 100 of 256 (39.1%) and 41 of 98 (41.8%) of clients with prediabetes and diabetes, respectively, had health insurance as well as a primary care clinician whom they had seen in the prior 12 months; a lack of health insurance was the most critical barrier to care engagement among both prediabetic and diabetic clients. There were substantially lower levels of care engagement among Latinx clients with prediabetes and diabetes, respectively, compared with non-Latinx clients with prediabetes.

#### Disclosure and Linkage Outcomes of Clients With Diabetes

Overall, 60 clients with diabetes (82.7%) were reached by CHWs within 24 hours for results disclosure and provided brief health education; 32 (53.3%) confirmed that it was a new diagnosis, 7 (11.7%) had a prior diagnosis but were not engaged in care, and 21 (35.0%) had a prior diagnosis and were engaged in care. Of 27 clients randomly selected for linkage outcome ascertainment, 21 (77.8%) were reached a median (IQR) 39 (36-42) days after undergoing diabetes testing (eTable 2 in the Supplement). Fifteen clients (71.4%) had follow-up primary care appointments scheduled, including 8 of 12 persons (66.7%) with a new diabetes diagnosis and 7 of 9 (77.8%) with a prior diagnosis; the median time to appointment after testing was 42 (27-56) days.

#### Multidisease Testing

Of 6591 clients who underwent rapid COVID-19 antigen testing, 385 (5.8%) were positive; persons who underwent hemoglobin A_1c_ testing were slightly more likely to test COVID-19 positive compared with those who declined hemoglobin A_1c_ testing (6.0% vs 5.8%; *P* < .001). Among clients with both rapid A_1c_ and COVID-19 results (883 individuals), the prevalence of COVID-19 did not differ according to A_1c_ classification: 32 (6.7%) in those with a normal A_1c_, 15 (5.0%) in those with prediabetes, and 6 (5.5%) in those with diabetes (*P* = .82). Of 598 clients who underwent rapid HIV testing, 2 (0.3%) were positive; however, upon disclosure it was confirmed that both had a prior HIV diagnosis.

### Implementation Outcomes

#### Acceptability

**Figure 3.**
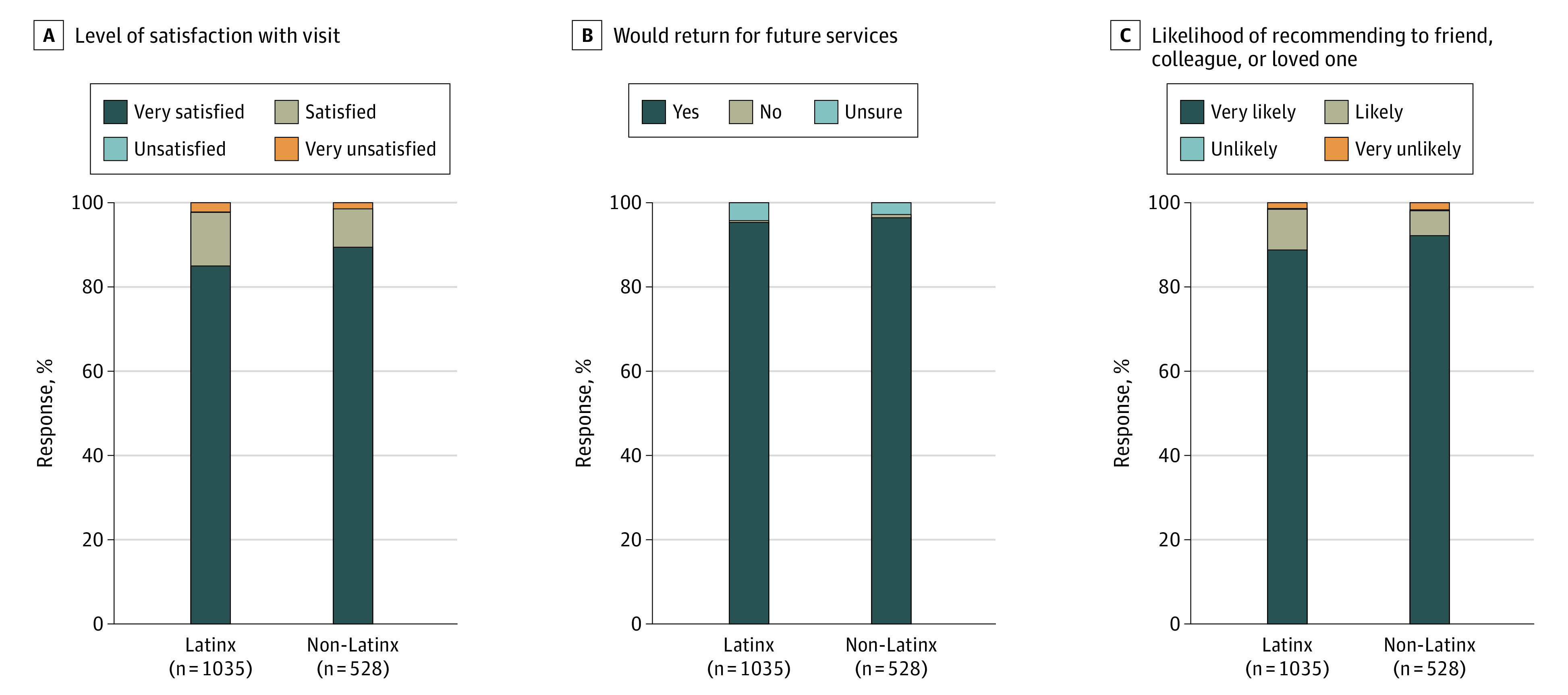
Measures of Acceptability and Satisfaction Among Clients Attending the Unidos en Salud Community-Based Multidisease Testing Site Stratified According to Ethnicity

Acceptability of the multidisease rapid testing program was very high among clients surveyed following their testing experience (1563 individuals) (Figure 3). Clients almost universally were either highly satisfied or satisfied with their visit (98.0%), were extremely likely to state they would return to the site for future services if offered (95.7%), and nearly all said they would recommend the multidisease testing site to a friend, colleague, or loved one (98.3%); these did not differ according to ethnicity. The features of the multidisease testing program that clients liked most included: friendly staff (41.1%), the fast and efficient process (28.7%), and the convenient location (10.2%). Latinx clients had greater preferences for the friendly (46.6% vs 31.6%) and bilingual (9.9% vs 1.7%) staff, while non-Latinx clients more strongly preferred the fast and efficient process (35.4% vs 24.8%) as well as its convenient location (14.9% vs 6.7%) (eFigure in the Supplement).

### Feasibility

The perceived feasibility of multidisease testing strategy was assessed among 21 Unidos en Salud staff members (eTable 2 in the Supplement). Overall, perceived feasibility of the multidisease testing program was high (composite mean [SD] score, 4.3 [0.5] of 5), with 19 of 21 (90.5%) staff members agreeing that it was feasible to integrate rapid hemoglobin A_1c_ testing into the existing community-based COVID-19 testing program. Perceived feasibility did not differ according to staff role or the amount of time an employee had worked at the site (eTable 3 in the Supplement).

## Discussion

In this study, we found that a strategy to make point-of-care diabetes testing available to low-income Latinx community members seeking COVID-19 testing at a community site in the Mission District in San Francisco was feasible to implement, highly acceptable to individuals tested, and reached our priority population of low-income Latinx persons not engaged in formal health care services. Among the more than 900 persons who underwent rapid hemoglobin A_1c_ testing during the 2-month evaluation period, 83% were Latinx, of whom more than 80% reported a household income of less than $50 000 and 54% that they were not engaged in care, despite the availability of a health coverage program in which all San Francisco residents are eligible regardless of documentation status. The program effectively identified a high proportion of people with prediabetes and diabetes—over a third of testers met criteria for prediabetes and 12% for diabetes. Notably, most of these community members had never been tested for diabetes before. This testing program was paired with education and linkage to care by CHWs, and 83% of participants received diabetes-related counseling from a CHW; in a subsample, 71% scheduled primary care follow-up. These data highlight the many unmet needs of low-income Latinx individuals and the critical need to identify innovative, effective, and sustainable strategies to surmount any preexisting access-related barriers to primary care for socioeconomically disadvantaged populations.

Our community-based COVID-19 and diabetes testing program represents a novel care engagement model beyond traditional brick-and-mortar health care services that could increase access to care for diabetes and other chronic diseases among socioeconomically disadvantaged populations. There were several design features crucial to our low-barrier testing approach that helped us successfully reach low-income Latinx participants (many of whom were first generation immigrants) and drove high levels of acceptability and satisfaction among the clients we served. This included a highly visible outdoor, neighborhood location that was conveniently located at a busy transportation and Latinx commercial and cultural hub and that was open on weekend days. Also, community members could drop in for free testing without a prior appointment or need to show identification at a site staffed by monolingual and bilingual Spanish speaking staff that were friendly and trained in good customer service principles. Furthermore, the site provided an efficient process using rapid point-of-care diagnostics with results available for COVID-19 and diabetes tests within an hour. While clients cited several reasons for getting tested for diabetes, the majority said that they came for COVID-19 testing, but that they might as well get tested for diabetes since they were already there. This suggests that an important additional feature driving the successful uptake of diabetes testing in this context was the act of colocating diabetes testing among other services for which there was significant community demand (eg, COVID-19 testing and vaccination).

Prior strategies seeking to expand access to diabetes education and testing outside of formal health care settings have predominantly used event-based approaches, in which intermittent screening events have been offered at churches, community centers, senior centers, shelters, shopping malls, events, and other community locations via both temporary infrastructure and mobile clinics.^15,16,17,26,27,28,29,30,31,32^ While event-based strategies may reach a relatively large number of individuals in a very short period, they provide limited opportunity for community members to become aware of and to physically access such services; they also may not provide sufficient opportunity to build community trust that can overcome the historical medical mistrust and immigration concerns of underserved communities marginalized by race or ethnicity. Community pharmacies have also been frequently been used to strategically expand the availability of diabetes testing and other health services to individuals not engaged in primary care^33,34,35^; however, due to structural barriers that hinder engagement in primary care, they remain inaccessible to many socioeconomically disadvantaged persons.^14,36,37^

As was observed in our study, recurrent community outreach and screening at culturally important locations have shown promise in other settings for engaging socioeconomically underserved populations in care for chronic diseases. For example, diabetes screening at Black-owned barbershops in Brooklyn identified a high prevalence (9%) of previously undiagnosed diabetes among Black Americans.^38^ Additionally, health education and diabetes screening regularly offered on Sundays at a *pulga* (flea market) in Texas found a diabetes prevalence of 25% among predominantly low-income Latinx individuals who were largely Mexican immigrants without health insurance.^17^ An important feature of their multicomponent strategy shared by the design of our testing strategy was the availability of a regular access point for testing; this allowed for predictable services and time for awareness-raising through word-of-mouth and peer referrals, as well as the development of community trust. However, it is crucial that novel community-based care engagement models for socioeconomically disadvantaged populations, especially those leveraging temporary pandemic-related public health responses, include features that facilitate reliable access and long-term sustainability. Potential considerations include integrating multidisease testing into weekly or monthly community events (eg, food distribution events) and the creation of free-standing, regularly staffed community health kiosks, which to date have largely focused on the provision of health education and measurement of vital signs.^39,40^

### Limitations

This study had several strengths, including that the multidisease testing strategy was developed in collaboration with community and public health partners and was tailored to specifically meet the needs of the local community. There were also some limitations to this study. Our multidisease testing strategy was conducted at a single site and leveraged community trust that we developed with the local Latinx community over a more than 15-month period during the pandemic; therefore, some of our findings may not be generalizable to other settings. Nonetheless, our site staff strongly felt that it was feasible to integrate point-of-care diabetes testing into an existing community COVID-19 testing site, and many communities have developed tailored COVID-19 testing and vaccination approaches to reach disadvantaged populations that could potentially be leveraged to implement a similar approach in other settings. Furthermore, despite reaching many low-income Latinx clients without linkage to primary care with hemoglobin A_1c_ testing, a majority of clients declined testing; reasons for declining hemoglobin A_1c_ testing were not directly elicited, but may reflect several factors, including knowledge of diabetes status through prior testing, perceived lack of time, and feeling too unwell to pursue additional testing, among others. Finally, linkage ascertainment was undertaken among a random subsample of clients. While these data are not fully generalizable because of their small sample size, this random sample provided insights on linkage. Although 71% had a follow-up appointment, the median follow-up time was 6 weeks; furthermore, the majority of uninsured clients did not have a follow-up primary care appointment at the time of the follow-up call. Additional research on the optimal strategies to overcome the multilevel barriers to care linkage following new diabetes diagnoses in community-based testing programs is urgently needed.

## Conclusions

In this health care improvement study of implementing rapid testing hemoglobin A_1c_ testing into an existing community-based COVID-19 testing program, we found that integrated, point-of-care testing reached and was acceptable to predominantly socioeconomically disadvantaged Latinx community members, was feasible to deliver, and resulted in the detection of a large number of community members with either prediabetes or diabetes—most of whom had never been tested for diabetes and lacked access to formal health care services. Leveraging public health responses to COVID-19 and future pandemics represents a highly promising but largely underexplored opportunity to improve health equity by engaging socioeconomically disadvantaged populations into care for diabetes, and this model has potential to expand testing and linkage for other highly prevalent chronic diseases.

## Notes

Supplement. **eTable 1.** Preexisting Diabetes Knowledge and Prior Testing and Diagnoses Among Participants Who Underwent Hemoglobin A1c Testing According to Ethnicity **eTable 2.** Characteristics and Linkage to Care Outcomes of Randomly Sampled Clients With Diabetes Diagnosed at the Unidos en Salud COVID-19 Community Testing Site in San Francisco, California, Between August 1 and October 5, 2022 **eTable 3.** The Perceived Feasibility of Integrating Rapid Hemoglobin A1c Testing Into an Existing COVID-19 Program Among Unidos en Salud Staff Members **eFigure.** Features of the Unidos en Salud Community-Based Multidisease Testing Site that Clients Said They Liked or Appreciated Stratified According to Ethnicity
