# Supplementary material for: Integrating Rapid Diabetes Screening Into a Latinx Focused Community-Based Low-Barrier COVID-19 Testing Program
Source: JAMA Netw Open. 2022 May 26;5(5):e2214163. doi: 10.1001/jamanetworkopen.2022.14163 (PMC9136625; doi:10.1001/jamanetworkopen.2022.14163)
Supplement: Supplement. — eTable 1. Preexisting Diabetes Knowledge and Prior Testing and Diagnoses Among Participants Who Underwent Hemoglobin A1c Testing According to Ethnicity eTable 2. Characteristics and Linkage to Care Outcomes of Randomly Sampled Clients With Diabetes Diagnosed at the Unidos en Salud COVID-19 Community Testing Site in San Francisco, California, Between August 1 and October 5, 2022 eTable 3. The Perceived Feasibility of Integrating Rapid Hemoglobin A1c Testing Into an Existing COVID-19 Program Among Unidos en Salud Staff Members eFigure. Features of the Unidos en Salud Community-Based Multidisease Testing Site that Clients Said They Liked or Appreciated Stratified According to Ethnicity [file jamanetwopen-e2214163-s001.pdf]

# Supplemental Online Content

Kerkhoff AD, Rojas S, Black D, et al. Integrating rapid diabetes screening into a Latinx focused community-based low-barrier COVID-19 testing program. *JAMA Netw Open.* 2022;5(5):e2214163. doi:10.1001/jamanetworkopen.2022.14163

**eTable 1.** Preexisting Diabetes Knowledge and Prior Testing and Diagnoses Among Participants Who Underwent Hemoglobin A1c Testing According to Ethnicity

**eTable 2.** Characteristics and Linkage to Care Outcomes of Randomly Sampled Clients With Diabetes Diagnosed at the Unidos en Salud COVID-19 Community Testing Site in San Francisco, California, Between August 1 and October 5, 2022

**eTable 3.** The Perceived Feasibility of Integrating Rapid Hemoglobin A1c Testing Into an Existing COVID-19 Program Among Unidos en Salud Staff Members

**eFigure.** Features of the Unidos en Salud Community-based Multidisease Testing Site that Clients Said They Liked or Appreciated Stratified According to Ethnicity

This supplemental material has been provided by the authors to give readers additional information about their work.

© 2022 Kerkhoff AD et al. *JAMA Network Open.*

**eTable 1.** Preexisting Diabetes Knowledge and Prior Testing and Diagnoses Among Participants Who Underwent Hemoglobin $A_{1c}$ Testing According to Ethnicity

| | Overall (n=429) | Latinx (n=347) | Non-Latinx (n=82) | p-value‡ |
|---|---|---|---|---|
| **Prior knowledge of diabetes** | | | | |
| High level of knowledge | 70 (18.7) | 58 (19.1) | 12 (17.1) | 0.35 |
| Some knowledge | 128 (34.2) | 102 (33.6) | 26 (37.1) | |
| Little knowledge | 101 (27.0) | 87 (28.6) | 14 (20.0) | |
| Never heard of diabetes | 75 (20.1) | 57 (18.8) | 14 (20.0) | |
| **Know someone with diabetes** | | | | |
| No | 122 (31.7) | 96 (30.5) | 26 (37.1) | 0.002 |
| Yes, family member | 140 (36.4) | 128 (40.6) | 12 (17.1) | |
| Yes, friend | 54 (14.0) | 41 (13.0) | 13 (18.6) | |
| Yes, family member and friend | 69 (17.9) | 50 (15.9) | 19 (27.1) | |
| **Prior testing for diabetes** | | | | |
| Yes | 129 (30.1) | 102 (29.4) | 27 (32.9) | 0.017 |
| No | 206 (48.0) | 177 (51.0) | 29 (35.4) | |
| Unsure | 94 (21.9) | 68 (19.6) | 26 (31.7) | |
| **Prior diabetes diagnosis** | | | | |
| Yes | 27 (6.4) | 21 (6.1) | 6 (7.4) | 0.31 |
| No | 302 (71.1) | 250 (72.7) | 52 (64.2) | |
| Unsure | 96 (22.6) | 73 (21.2) | 23 (28.4) | |

‡P-value represents Chi-squared or Fisher's exact test for comparison of proportions.

© 2022 Kerkhoff AD et al. *JAMA Network Open.*

**eTable 2.** Characteristics and Linkage to Care Outcomes of Randomly Sampled Clients With Diabetes Diagnosed at the Unidos en Salud COVID-19 Community Testing Site in San Francisco, California, Between August 1 and October 5, 2022**.**

| Client | Hemoglobin A1c level | Diabetes diagnosis | Has health insurance | Has primary care | Referral made to primary care | Follow-up appointment scheduled | Days from diabetes test to follow-up appointment | Follow-up appointment attended |
|---|---|---|---|---|---|---|---|---|
| **Able to reach client for linkage outcome ascertainment** | | | | | | | | |
| 1 | 6.7 | New | No | No | Yes | No | - | - |
| 2 | 6.5 | New | No | No | Yes | Yes | 52 | Unknown |
| 3 | 7.3 | New | No | No | Yes | No | - | - |
| 4 | 7.3 | New | No | No | Yes | No | - | - |
| 5 | 7.5 | New | No | No | Yes | No | - | - |
| 6 | 7.3 | New | Yes | No | Yes | Yes | 56 | Yes |
| 7 | 7.5 | New | Yes | Yes | No | Yes | 55 | Unknown |
| 8 | 7.3 | New | Yes | Yes | No | Yes | 62 | Yes |
| 9 | 7 | New | Yes | Yes | No | Yes | 23 | Unknown |
| 10 | 13 | New | Yes | Yes | No | Yes | 31 | Unknown |
| 11 | 6.6 | New | Yes | Yes | No | Yes | 7 | Yes |
| 12 | 7.1 | New | Yes | Yes | No | Yes | 30 | Unknown |
| 13 | 6.8 | Known | No | No | Yes | No | - | |
| 14 | 6.7 | Known | Yes | Yes | No | Yes | Unknown | No |
| 15 | 6.7 | Known | Yes | Yes | No | Yes | 27 | Yes |
| 16 | 6.7 | Known | Yes | Yes | No | Yes | 16 | Yes |
| 17 | 7.7 | Known | Yes | Yes | No | Yes | 86 | Unknown |
| 18 | 7.6 | Known | Yes | Yes | No | Yes | 112 | Unknown |
| 19 | 13 | Known | Yes | Yes | No | Yes | Unknown | Yes |
| 20 | 7 | Known | Yes | Yes | No | Yes | 42 | Unknown |
| 21 | 6.5 | Known | Yes | Yes | No | No | - | - |
| **Unable to reach client for linkage outcome ascertainment** | | | | | | | | |
| 22 | 6.5 | New | Yes | Yes | No | Unknown | - | - |
| 23 | 6.8 | New | Yes | Yes | No | Unknown | - | - |
| 24 | 7.1 | New | Yes | Yes | No | Unknown | - | - |
| 25 | 8 | Known | Yes | Yes | No | Unknown | - | - |
| 26 | 6.7 | Known | Yes | Yes | No | Unknown | - | - |
| 27 | 9.6 | Known | No | No | Yes | Unknown | - | - |

© 2022 Kerkhoff AD et al. *JAMA Network Open.*

**eTable 3.** The Perceived Feasibility of Integrating Rapid Hemoglobin A1c Testing Into an Existing COVID-19 Program Among Unidos en Salud Staff Members

Staff members completed a brief paper-based survey of four, five-point Likert scale questions, where 1=Completely disagree, 2=Disagree, 3=Neither Agree nor Disagree, 4=Agree, and 5=Completely Disagree. Therefore, the higher the mean value, the more feasible integration of rapid hemoglobin A1c testing into existing COVID-19 testing infrastructure was felt to be. All mean values were out a possible score of 5.

| | **Proportion who completely agree/agree (%)** | **Mean value (SD)** |
|---|---|---|
| **Feasibility of Intervention Measure (n=21)** | | |
| 1. Screening/testing for chronic diseases in persons getting tested for COVID-19 is implementable. | 85.7% | 3.9 (1.2) |
| 2. Screening/testing for chronic diseases in persons getting tested for COVID-19 is possible. | 100% | 4.5 (0.5) |
| 3. Screening/ testing for chronic diseases in persons getting tested for COVID-19 is doable. | 100% | 4.2 (0.5) |
| 4. Screening/ testing for chronic diseases in persons getting tested for COVID-19 is easy. | 85.7% | 4.2 (0.7) |
| **5. Overall composite feasibility*** | **90.5%** | **4.3 (0.5)** |
| **Staff role** | | |
| Testing (n=9) | 100% | 4.4 (0.5) |
| Registration (n=6) | 83.3% | 4.2 (0.4) |
| Clinical and care engagement support (n=4) | 75.0% | 4.1 (0.5) |
| Other (n=2) | 100% | 4.5 (0.7) |
| **Length of time worked at site** | | |
| <3 months (n=6) | 100% | 4.2 (0.2) |
| ≥3 months (n=15) | 86.7% | 4.3 (0.5) |

*Determined by calculating each individual respondent's mean composite score across four individual feasibility questions. Those with a mean composite score ≥4.0 were considered to agree. The overall mean value was calculated using the mean of all individuals' means.

© 2022 Kerkhoff AD et al. *JAMA Network Open.*

**eFigure.** Features of the Unidos en Salud Community-based Multidisease Testing Site that Clients Said They Liked or Appreciated Stratified According to Ethnicity

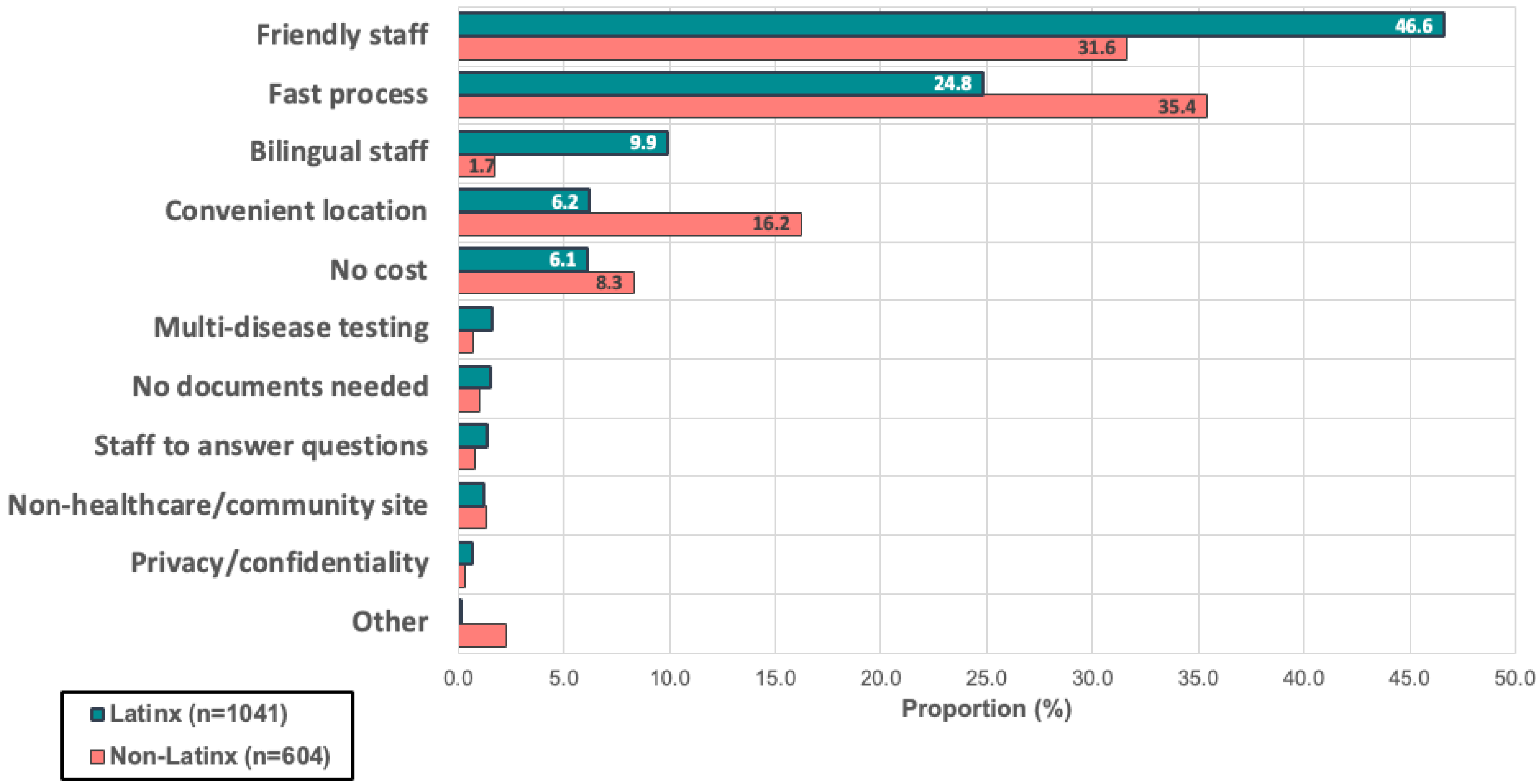


© 2022 Kerkhoff AD et al. *JAMA Network Open.*
